# A Novel Weighted Clustering Algorithm Supported by a Distributed Architecture for D2D Enabled Content-Centric Networks

**DOI:** 10.3390/s20195509

**Published:** 2020-09-25

**Authors:** Saad Aslam, Fakhrul Alam, Syed Faraz Hasan, Mohammad Rashid

**Affiliations:** Department of Mechanical & Electrical Engineering, SF&AT, Massey University, Auckland 0632, New Zealand; f.alam@massey.ac.nz (F.A.); f.hasan@massey.ac.nz (S.F.H.); m.a.rashid@massey.ac.nz (M.R.)

**Keywords:** D2D communication, distributed architectures, 5G, content-sharing, clustering algorithm, content-centric networking, network virtualization

## Abstract

Next generation cellular systems need efficient content-distribution schemes. Content-sharing via Device-to-Device (D2D) clustered networks has emerged as a popular approach for alleviating the burden on the cellular network. In this article, we utilize Content-Centric Networking and Network Virtualization to propose a distributed architecture, that supports efficient content delivery. We propose to use clustering at the user level for content-distribution. A weighted multifactor clustering algorithm is proposed for grouping the D2D User Equipment (DUEs) sharing a common interest. The proposed algorithm is evaluated in terms of energy efficiency, area spectral efficiency, and throughput. The effect of the number of clusters on these performance parameters is also discussed. The proposed algorithm has been further modified to allow for a tradeoff between fairness and other performance parameters. A comprehensive simulation study demonstrates that the proposed clustering algorithm is more flexible and outperforms several classical and state-of-the-art algorithms.

## 1. Introduction

Unprecedented demand for multicast applications is driving a move towards content-centric cellular networks. However, to accommodate such services, future networks must go beyond the capabilities of their current generation counterparts [1,2]. Cellular users are actively engaged in generating and sharing the content of various types [3]. Therefore, future networks will require to handle significantly higher multimedia services [4,5]. Existing centralized architectures and mechanisms may not be able to meet the content-sharing demands [2,3]. Decentralized mechanism and load mitigation in cellular networks are required to meet the rising demand of content sharing. One of the techniques that can effectively address load mitigation is device-to-device (D2D) communication [5]. It is different from the conventional cellular communication where all communication goes via the core network irrespective of proximity of the devices [6]. Various architectural frameworks have been proposed in the literature to support D2D-based content sharing. The concept of clustering users in proximity sharing a common interest has been very popular for multicasting scenarios [3]. Typically, an intermediate node, termed as cluster head (CH) fetches the content from the Base Station (BS) and delivers it to several content requestors [7,8,9]. Medium Access Control (MAC) strategies utilizing clustering for reducing energy consumption specific to multicasting scenarios are being proposed [8,9,10]. It is reported in the literature that clustering improves the D2D caching efficiency and communication [11,12]. We presented the effectiveness of utilizing clustering in a D2D multicasting scenario in one of our previous works [13]. Formation of appropriate clusters is significant as it influences the performance of the underlay cellular networks [3]. Considering all these, we propose a multifactor weighted clustering algorithm in this article. The algorithm utilizes proximity, channel gain, and channel variance (details in later sections) to form the clusters.

To realize clustering that supports content-sharing via D2D, a suitable architecture is necessary that not only conforms to the standards of future cellular networks but is also distributive in nature. Moreover, it should be capable of handling a high user density. Therefore, we propose a decentralized architecture for content-sharing that is suited for 5G and future cellular network. This architecture utilizes Content-Centric Networking (CCN) and Network Virtualization (NV), key technologies of 5G architecture [14,15,16,17,18,19]. Our proposal also employs the frame structure utilized in the published article on 5G [20,21,22,23]. The details regarding the proposed architecture can be found in Section 3.

Recent literature suggests that the geographical distribution of mobile users play a vital role in successful content-caching [24]. Moreover, to ensure nearby availability of content, caching at a D2D device should consider social ties and requests pattern. In a real-scenario, different social events such as sporting events and concerts can significantly influence the clustering schemes and the multicasting scenario. Consider the example of a concert where users are interested in the same videos of the artist. In such cases where users share a strong social relationship, a user (e.g., CH) can easily contribute to the distribution of the video. Therefore, it is important to identify users with common social characteristics. In this study, the proposed decentralized architecture is supported by simple hash-based functions that have been previously used in multimedia broadcast networks for identifying users with a common interest.

The major contributions of this research work are as follows:

- A distributed architecture is proposed that is effectively supported by hash functions to identify the socially connected users. This is in contrast to the majority of the published works on D2D multicasting that do not consider distributed architecture along with content-identification.
- A novel multifactor weighted clustering has been proposed. The performance of the proposed algorithm is shown to be superior compared to five benchmarked algorithms. In addition, the weights of the algorithm can be adjusted to suit the system’s requirements. This flexibility in trading off the performance with respect to various parameters is not available for existing algorithms.
- The benchmarked algorithms are tested for throughput fairness which has not been reported in the literature on clustering. Moreover, different from the existing works, the impact of the number of clusters on the energy consumption and area spectral efficiency is also demonstrated.
- To the best of the author’s knowledge, reported work in the literature considers either the spatial distribution of users or users’ social ties for their respective clustering algorithms. We propose to include both to make the clustering process comprehensive and evaluate its impact on the system’s performance.

The rest of the manuscript is organized into five sections. Relevant literature and related work have been summarized in the next section. Section 3 presents the details of the proposed architecture and content identification technique. The proposed clustering algorithm is described in Section 3 as well. Section 4 presents the system model including the simulation setup. It explains all the major assumptions of the simulation environment, and mathematical models of the performance parameters. Section 5 discusses the performance of the proposed scheme benchmarked against existing methods. The findings of this research work are summarized in the Conclusion section while discussing the future directions of the proposed study.

## 2. Related Work

With exponentially increasing network devices, it is becoming difficult to fulfill the QoS requirements of multimedia services [25,26]. The growth of cellular devices has been addressed with the concept of dense networks having a large number of small cells. However, the limited capacity of the backhaul becomes a bottleneck in such a scenario [27]. It has been proposed that the significant growth in 5G networks and beyond can be accommodated by investing more in Content-Centric Networks (CCN). The notion behind a CCN is to present a scalable and efficient mechanism for content delivery [28]. The techniques developed around CCN are expected to reduce the transmission delay by caching the data within the network. Data caching is performed closer to the group of mobile devices/content requesters by exploiting social ties (e.g., shared interest in content) or connections [11,29]. Device-level caching can be facilitated by D2D communication. D2D has been effectively used to disseminate data in various network scenarios. However, the network architectures presented in many of these works [11,29,30,31] considering the D2D multicasting scenario are centralized in nature and require massive message passing to make the whole scheme work. Such schemes do not meet the requirements of dense future cellular networks. Contrary to this, we have proposed a decentralized architecture that can effectively support the D2D multicasting scenario.

Researchers have investigated the technologies that address the scalability of the network and the ability to cater to the growth in wireless traffic and services. Network virtualization concept has been widely used [27,28,29]. Virtual Private Networks (VPN) is one of the examples. Network virtualization aims to slice the resources of cellular architecture into virtual resources to be shared among multiple users. It should be noted that by cellular resources we mean a licensed spectrum and infrastructure e.g., Core Network and Radio Access Network, etc. [29]. All the signaling and message passing that needs to take place to set up the virtual network is well researched and presented in the literature [27,28,29]. Our study proposes a network architecture that combines the concepts of CCN and NV. This is different to the schemes found in literature, as we merge both the technologies. There are significant advantages to the proposed merger. One of the most important features is that not only the cellular resources but the actual contents can be shared. Duplicate transmissions exhaust the cellular resources. The content-sharing among the networks with the aid of virtualization can significantly reduce these redundant transmissions. Details of the architecture are presented in the next section.

Clustering complements the proposed architecture. However, when a multicasting scenario is considered in the literature within the context of clustering, most works assume that users sharing a common interest have already been identified. Furthermore, if a system model is presented, how a typical cellular architecture can support such a model is not shown or glossed over. Most articles either discuss social tie/social interest modelling or clustering algorithm in detail but not both [32,33]. In [32], a clustering algorithm has been proposed and social metric is also considered as an important factor. However, it does not provide any details around how the users having the same interest are identified. The architecture that supports their system model is not presented either. Research work presented in [33] discusses the social ties/social attributes in detail and effectively describes the mechanism behind modelling social metrics. It also considers clustering for a multicasting scenario but the clustering itself is assumed to have taken place by placing the users in a certain grid. Therefore, clustering and its effects on the performance parameters have not been discussed. Moreover, it is important to note that [32,33] uses centralized mechanisms. In contrast to these works, we chose to address both content-identification (through the hashing concept) supporting a decentralized architecture and a clustering algorithm to complete the big picture.

The consideration of distributed architecture, different performance parameters, and the flexibility to trade-off the performance for fairness is not seen in the recent and relevant literature. Different research works mentioned in Table 1 show the stated fact. For instance, a few works focus on throughput, whereas others provide details on energy consumption. On the other hand, only a few articles have considered Area Spectral Efficiency (ASE) and Fairness. Most importantly, all these works rely on centralized architectures. As opposed to this, our work provides the details on all these parameters while proposing a distributed architecture.

## 3. Proposed Distributed Architecture and Clustering Algorithm

**Figure 1:**
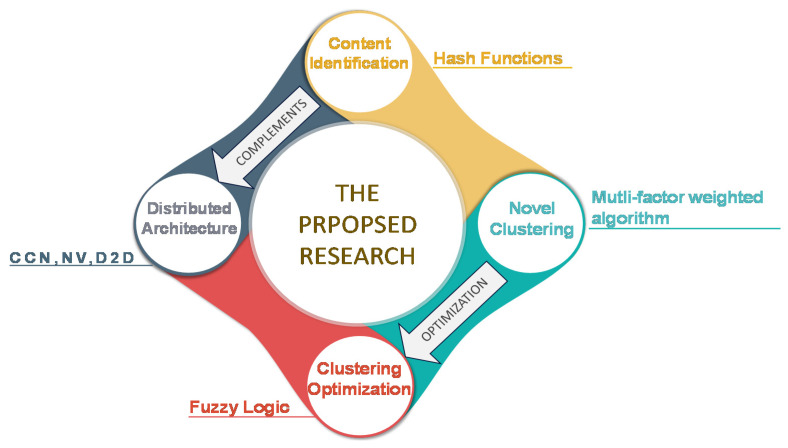
Summary of the Proposed Mechanism. The four critical aspects are: distributed architecture implementation using Content-Centric Networking (CCN) and Network Virtualization (NV), identification of users with common interest using hash functions, cluster formation with the proposed multifactor algorithm and optimizing the clustering algorithm using fuzzy logic.

The proposed concept is illustrated in Figure 1. The utilization of clustering for content-sharing is the key concept of the proposed architecture. The architecture is based on CCN and NV. Hash functions identify users with common interest who are then organized in clusters based on the proposed multifactor algorithm. The clustering algorithm is optimized using fuzzy optimization which is useful in optimizing clustering as well as other parameters of a cellular network [43,44,45,46]. Each cluster has a cluster head (CH) that are responsible for multicasting the required information to the cluster members. After clustering, all the content-requests from any given cluster traverse through the CH and users are served via the CH utilizing D2D communication.

The concept of CCN is predicated on the requested content reaching the requester without having the need to reach the content publisher/provider [29]. Therefore, caching the requested content at an intermediate node will enable the content-delivery with reduced energy consumptions and latency. Once the intermediate node has cached the content, it can be provided to several requesters.

**Figure 2:**
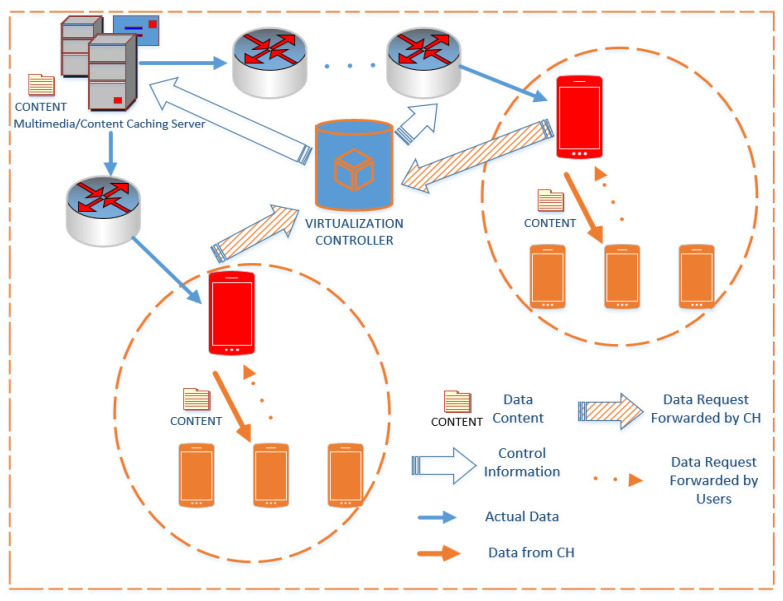
The proposed distributed network architecture. Each red device represents a cluster head that forwards the requested content to the virtualization controller that connects with the multimedia servers to fetch the contents.

Architectures supporting Content-Centric Networking have been proposed in the literature [28,29,47,48]. However, these works consider centralized mechanisms. The architectures presented in [28,29] involve BS, and significant signaling is required to take place between BS and the D2D nodes, before the content-delivery. On the other hand, [47] does not provide any details on the architecture. The work presented in [48] does consider decentralized mechanisms, but it does not explicitly show any architecture that supports their mechanism. Our research considers a similar approach as presented in [28,29] with necessary modifications to accommodate the clustering of users for content-sharing scenarios and making the scheme distributive. Figure 2 shows the network model of the proposed content-centric architecture. It is different from a conventional wireless network such as Internet Protocol (IP). The basic difference lies in the establishment of the connection. IP based networks first establish the connection between the requestor and the provider before the content is delivered. In contrast, the content is requested without the establishment of the connection with the host/content-provider in CNN. The proposed architecture utilizes Content-Centric Networking as well as network virtualization. The controller for virtualization, shown in Figure 2, is responsible for providing the location of the content-holder as well as setting the virtual infrastructure components for content-delivery. A mobile user requests certain content without the information of the host which holds that content. Another important entity of the proposed architecture is the caching server. It is an integral part of the network which caches popular contents and reduces duplicate transmissions.

**Figure 3:**
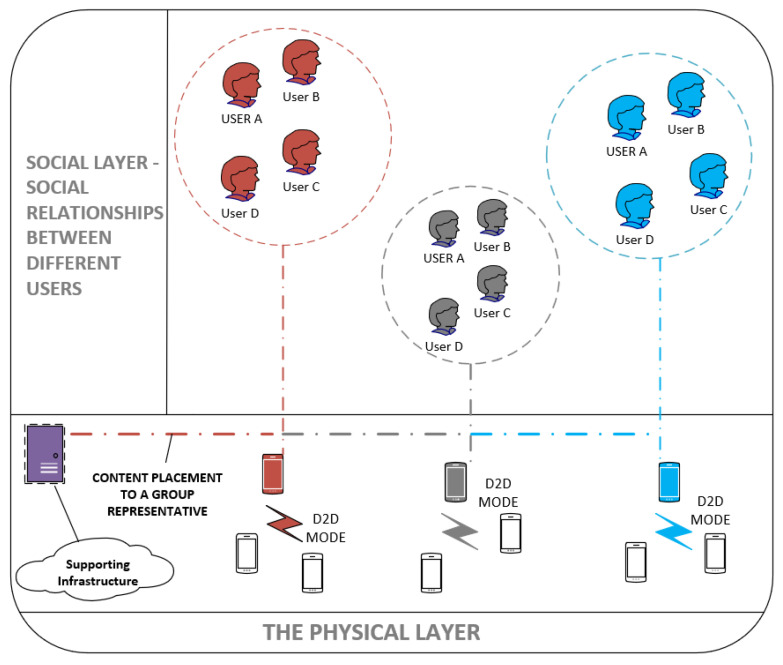
The visualization of a layered network showing the interaction between the social users and the corresponding physical layer.

Figure 3 shows the layered network. The first layer, termed as a social layer, represents the social ties that exist among different groups of users. The physical layer represents the mobile devices that model the communication taking place in various clusters represented by a CH. It also shows the supporting infrastructure required to set up the communication and making the content-delivery possible.

### 3.1. Content Identification Using Hash Functions

One of the important aspects of the proposed architecture is the mechanism that addresses the content-identification. We propose to utilize hash functions [49] for content-identification. Hash functions perform the mapping between the given data and hash of a specific length. The size/length of the output of a hash function does not depend on the length of the input. Hash can be regarded as a ”signature” for a given text [49,50,51]. One of the major applications of hash functions lies in the field of multimedia broadcast networks, as a content identifier [49,50,51]. The hash function aids the network by providing content identification to easily determine which content has been broadcasted, timing information, and to what station. Several hashing algorithms exist in the literature; we suggest using SHA-256 due to its reduced complexity and speed [52,53].

**Figure 4:**
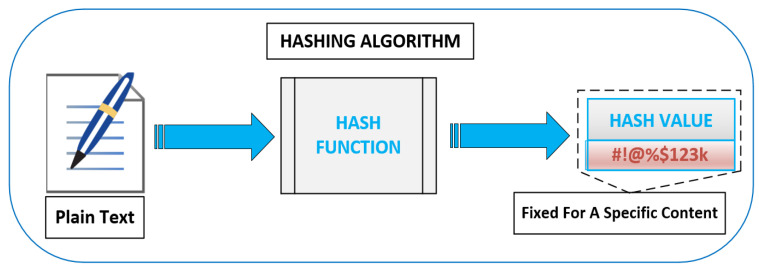
The hash function: plain text to hash value.

The binary sequence generated by the hash functions for a particular ”text” or ”name” will always be the same as shown in Figure 4. Therefore, if we produce the hash of the different contents at the content-servers, the hash value can be matched with the one generated by the content requestors. A match means that the same content is being demanded. Groups requesting the same content can, therefore, be identified based on the hash values. It is clear that hashing will not only help in identifying the content but also the group of users sharing the same interest. Therefore, we believe that hashing is a natural choice.

### 3.2. The Proposed Clustering Algorithm

**Figure 5:**
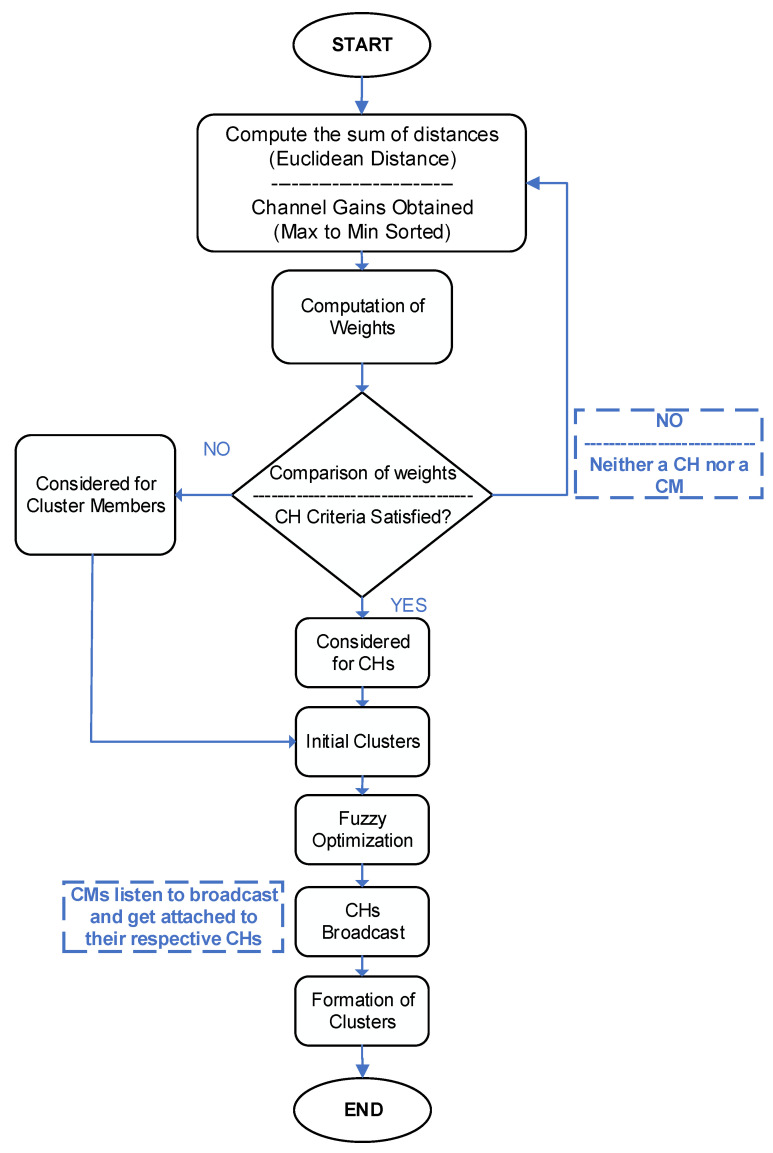
Flow chart of the proposed clustering algorithm.

Clustering commences once users demanding the same content have been identified. The user clustering process consists of three main steps: selection of appropriate clustering metrics, identification of the devices suitable for being a CH, and finally, associating the cluster members with their respective CHs. The overall clustering process is shown in the flow chart of Figure 5.

#### 3.2.1. Weighted Clustering Approach

CHs are selected on a per-frame basis. The duration of one frame is 10 ms following the relevant literature. All the users are considered CHs for the first frame; therefore, the clustering algorithm is implemented for the next frames. It is assumed that every node is capable of being a CH and has enough energy [12]. The position of the nodes remains the same during one frame. However, for the next frame user distribution/placement of users change and, therefore, every simulation represents a different user distribution. This is in accordance with the standard literature relevant to multimedia multicasting scenarios [11,12].

After the initialization, the algorithm gathers the information about the clustering metrics and clusters are formed, details of which can be found in the subsequent sections. Before the clustering takes place, the distance among the devices and the channel conditions are obtained and conveyed to all the neighbors during the discovery phase as explained in the next subsection. Based on the information received, CHs announce its cluster members. It is assumed that multiple users can be detected simultaneously by the CH. All the members listen to the broadcast of the CHs and get attached to the one that serves them the best considering distance and channel conditions.

#### 3.2.2. Device Discovery

Though the device discovery is out of the scope of this research, we utilized the information obtained through the device discovery. Therefore, the authors are describing the discovery process within the context of the proposed algorithm.

Before the clustering takes place, it is necessary to discover the devices and create a neighbor list. We assume, as is common practice in the literature [54,55,56,57,58], that the neighbor list is available with the nodes. For these tasks, we propose to use the Peer Discovery Resource (PDR). PDR represents a resource unit, used to transmit the discovery signal or beacon signal. Two of the standard PDR structures that are used in published literature are LTE-A and FlashlinQ. Literature suggests that a considerable amount of information can be conveyed using either of these structures [34,56,57]. Moreover, different research works have utilized the PDR to send the clustering-related information [55,56]. We propose to utilize the same concept and use the PDR to send the information regarding the predefined clustering metric detailed in the subsequent section. Therefore, the signaling load for the proposed clustering scheme will be accommodated by standard signaling taking place in a D2D network.

The users in proximity to one another receive the discovery signals. Devices decode this signal containing information such as device or user ID and its link characteristics (such as SINR, channel conditions) with the user. Based on these characteristics, a device decides which of the users whose signal it has received can be classified as neighbors. There are various advanced channel estimation algorithms and processes for 5G networks [58] that can be utilized for this purpose. Once the neighbor detection has taken place, every user possesses the list of its neighbors.

#### 3.2.3. Clustering Metrics

The selection of clustering metrics significantly impacts the system’s performance. The two factors of the proposed clustering algorithm include distance and channel conditions among the users. Each factor is assigned to its respective weight. These metrics are selected due to their effect on target performance parameters such as throughput, area spectral efficiency, and energy consumptions. The significance of this selection will be further highlighted in the results section.

1. The Distance among the Nodes

Recent literature has identified the significance of the spatial distribution of users as it directly influences the caching efficiency [24]. Furthermore, there is a high probability of successful D2D transmission if the devices are in proximity [11]. Hence, we chose distance among the nodes as an important metric for forming appropriate clusters. It is also important since users that do not exist in proximity are not ideal candidates for being a part of the same cluster even with a strong social relationship.

2. Channel Conditions

Since we are considering a multicasting scenario where a CH will be communicating with the rest of the cluster members, it is important for the cluster members to have good links with the CH. If we ignore these conditions, both inter-cluster and intra-cluster communication might be impaired. Therefore, we believe that channel conditions between the prospective CH and its cluster members is an important metric.

#### 3.2.4. Cluster Head Selection

During the CH selection process, devices use PDR to broadcast beacons continuously. These beacons include a predefined metrics (e.g., distance, channel conditions). Every device decoding the beacon stores the corresponding metric and its identifiers (see Section 3.2.2). This information is vital for the devices to select a CH and delegating the control to it for further communication. If a certain device is not able to receive a beacon signal, it might be out of reach of another device, and it can self-select itself as CH. Once the metric information is received from the beacons, all the devices compare their metrics. The devices with the lowest metric values are identified as CHs. The remaining becomes the cluster members. It should be noted here that all the users need to fulfill the predefined criterion to be considered for clustering. It is based on the social relationship among the devices. The following steps summarize the proposed algorithm.

Step 1: Determine the neighbors of each node using D2D discovery. Parameters of interest are stored.

Step 2: Determine the nodes sharing the same interest/content using the hash function.

Step 3: Compute the sum of distances (Euclidean Distance) for all the nodes against all their neighbors.

(1) $$D_{\left(a,b\right)}=\;\sqrt{(a_{X}{}^{2}-\;b_{X}{}^{2})+\;\left(a_{Y}{}^{2}-\;b_{Y}{}^{2}\right)}$$

where *a* and *b* represent any two neighboring devices.

Step 4: Weights of the nodes are accumulated as follows:

(2) $$W_{T}=I.\left[w_{1}*D_{\left(a,b\right)}+\;w_{2}*\left(\frac{1}{h_{ab}}\right)\right]$$

$w_{1},\;w_{2}$ represents the weights given to distance, channel gains, respectively. $h_{ab}$ represents the channel gain between the nodes a and b. The weights represented in Equation (2) are such that $\sum_{f=1}^{2}w_{f}=1$. The node with the minimum $W_{T}$ is chosen as the CH.

Since only nodes sharing a common interest should be considered for clustering, the total weight is being multiplied with a binary interest-factor denoted by “I”, so that if $z=\;w_{1}*D_{\left(a,b\right)}+w_{2}*\left(\frac{1}{h_{ab}}\right)$, then,

(3) $$W_{T}=\;\{\begin{array}{c} z,\;\;\;\;\;I\neq 0\\ 0,\;\;\;\;\;I=0 \end{array}\}$$

Step 5: Compare the weights for each node and select the cluster head corresponding to the smallest $W_{T}$.

Step 6: For the remaining devices, repeat steps 3 and 4, until each node is either selected as a CH or a CM.

Step 7: Clustering optimization using Fuzzy (details in Section 3.2.6).

#### 3.2.5. Feature Scaling for the Clustering Metric

The two different factors for clustering (Equation (2)) do not have the same range of values. Therefore, data normalization was performed [59]. There are different normalization techniques available in the literature such as Min-Max normalization, decimal scaling, and Z-score scaling [59,60]. Min-Max and decimal scaling do not handle outliers very well. Therefore, we chose to utilize the z-score normalization given by Equation (4).

(4) $$Z_{score}=\;\frac{x-\;\bar{x}}{\sigma }\;$$

where x is the original value (e.g., channel condition) for which we calculated the z-score, and $\bar{x}$ and σ represent the mean and the standard deviation of x, respectively.

#### 3.2.6. Fuzzy Optimization of Clustering

The initial clusters formed based on the proposed algorithm need to be optimized. Therefore, a fuzzy optimization technique was applied. Fuzzy optimization partitions the users into C clusters based on the proposed criterion of clustering. Each input to this function is attached to an attribute such as the weights in our study. Fuzzy optimization is based on a partition matrix [61] P ∈ $Q_{FO}$ where;

(5) $$Q_{FO}=\;\{P\;\in \;{\left[0,1\right]}^{C*N}\;|\overset{c}{\underset{i=1}{\sum }}p_{ik}=1,\;k\;1,\;.\;.\;.N,\;\overset{N}{\underset{i=1}{\sum }}\;p_{ik}>0,\;\;i=1,\;.\;.\;.\;c\;\}$$

The objective function of Fuzzy Optimization is given by Equation (6) [61]:

(6) $$OF\;\left(P,\;V\right)=\overset{c}{\underset{i=1}{\sum }}\sum_{k=1}^{N}p_{ik\;}\|X_{k}-V_{i}\|{}^{2}$$

**Figure 6:**
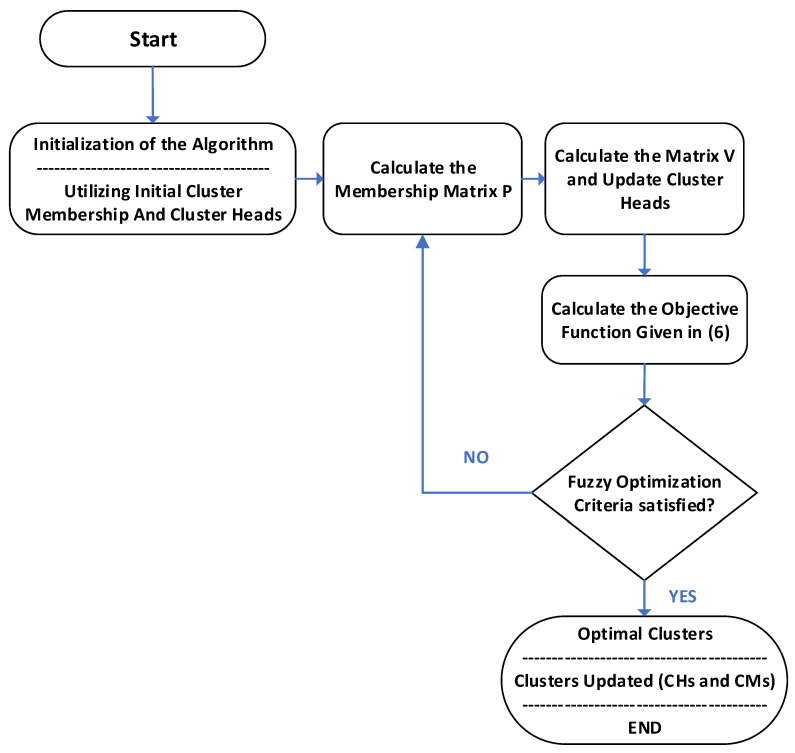
Optimization flow chart.

Equation (6) represents the objective function where C is the number of clusters and V is the set of cluster centers. N represents the number of samples (users in our case) and $X_{k}$ is the kth calculated sample where ${\left\|.\right\|}^{2}$ represents the Euclidean norm, and $p_{ik\;}$ denotes the membership of $X_{k}$ to cluster i. Each element of the partition matrix is a measure of the extent to which a particular user belongs to a certain cluster. The complete optimization process is explained in the flow chart given in Figure 6.

#### 3.2.7. Communication

**Figure 7:**
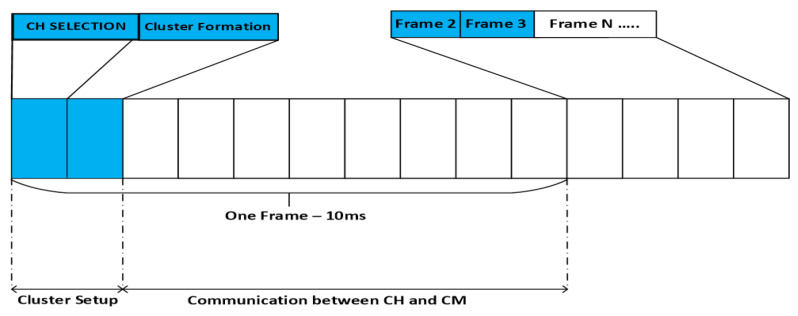
Frame structure for clustering.

After the selection of CHs, they broadcast a message containing their IDs. They can use the same PDR used for neighbor detection to broadcast the results once the broadcast is received, and all the non-CH devices select those CHs to which they are closest and receive better channel conditions. The cluster members then associate themselves with a certain cluster, and the formation of the clusters is complete. The above-mentioned procedure is completely decentralized which is very important for dense networks. Once the clusters are formed, communication of all the cluster members goes via the CH. The operating phases of the proposed algorithm are shown in Figure 7. The next frame follows the same activities.

## 4. System Model and Simulation Setup

We consider a single cell where users are randomly distributed. In-band D2D communication using the underlaying concept is considered. In this case, D2D reuses cellular resources. These techniques are well researched [12,62,63]. The reason for considering the underlaying concept is that reutilizing the resources improve spectral efficiency. However, it creates interference and, therefore, was considered in our simulation scenario. Conventionally, BS provides the requested content; however, it comes at the expense of increased energy consumption and usage [62,63]. In contrast, the CH is responsible for delivering the contents to the requestors as depicted in Figure 2. Once the data have been fetched by the CH, the requested content is distributed utilizing the D2D multicast communication.

### 4.1. Mathematical Models for Performance Parameters

#### 4.1.1. Achievable Rates for Cluster Head and Cluster Members

There are total N users in the network which constitute the set $N\;=\;\left\{m_{1,\;}\;m_{2},\;m_{3-\;-\;-,\;}m_{N}\right\}.$ The CHs and cluster members are indexed as j and k, respectively. For clarity, all the other symbols are summarized in Table 2.

The achievable rate at the CH can be written as:

(7) $$R_{CH_{j}}=B\log_{2}\left(1+SNR_{CH_{j}}\right)$$

where the SNR of the $CH_{j}$ is given by:

(8) $$SNR_{CH_{j}}=\;\frac{P_{B}\;h_{BS,\;CH_{j}}}{N_{o}\;B}$$

Therefore, we may write Equation (7) as:

(9) $$R_{CH_{j}}=B\log_{2}\left(1+\frac{P_{B}\;h_{BS,CH_{j}}}{N_{o}B\;}\right)\;$$

Since we are considering a multicasting scenario, the achievable rate depends on the worst physical link. Otherwise, the successful reception of the content for all cluster members cannot be made certain. Therefore, the achievable rate at the cluster member $m_{k}$ can be written as follow:

(10) $$R_{m_{k}}=B\log_{2}\left(1+\frac{P_{CH_{j}}h_{m_{k},CH_{j}}}{N_{o\;}\;B}\right)$$

It should be noted that $R_{m_{k}}$ is the minimum achievable rate to make sure that all the cluster members receive the content.

#### 4.1.2. Energy Model

Downlink energy consumption is considered in this study. We utilized the energy consumption model presented in [64]. We assumed that the content demanded by the users is a file of size $“F_{S}”\;bits.$ Suppose this file needs to be transmitted from $CH_{j}$ to cluster member $m_{k}$ with an achievable data rate of $R_{m_{k}}$. The time required to transmit this file is $\left(\frac{F_{S}}{R_{m_{k}}}\right)seconds$. Therefore, energy consumption $E_{C}$ in one of the clusters “C” can be written as:

(11) $$E_{c}=\;\frac{F_{s}P_{chrx}}{R_{CH_{j}}}+\;\frac{F_{s}P_{CH_{j}}}{R_{m_{k}}}+\sum_{\begin{array}{c} j\neq m\\ \forall m \end{array}}\frac{F_{s}P_{mrx}}{R_{m_{k}}}\;$$

Equation (11) represents the sum of three independent terms. Energy consumption of CH to receive data is represented by the first term, whereas the second term represents the energy consumed by the CH to transmit the data to their cluster members. The sum of the energy consumed by the cluster members to receive the demanded content is shown by the last term in Equation (11).

### 4.2. Simulation Setup

The simulation environment was built on MATLAB. We considered a network model similar to Figure 2. A single cell of 1 sq.km area was considered. For the conventional cellular communication scenario, the BS was placed at the center of the cell. Moreover, it is important to mention that we explored the performance of a multimedia application (content). The packet size is 100 kB as suggested by relevant literature [65]. This simulation can easily be extended for any other multimedia application (e.g., video broadcast, eHealth, etc.) by varying the file size and packet interarrival rates [65]. We selected the weights empirically, which can be adjusted according to the system requirements. The number of clusters formed to produce all the results were chosen using the Calinski–Harabasz criteria [66]. Various user densities have been considered to produce the results. The optimum number of clusters for various user densities is different, and hence, a specific number is not explicitly mentioned. All the simulation parameters of interest are detailed in Table 3. Parameters related to channel and energy consumption are adapted from the relevant literature [64,67,68].

## 5. Results and Discussion

### 5.1. Impact of Clustering and Social-Interest

The proposed algorithm takes clustering and social interest into account, as both have a significant impact on the system. To demonstrate this impact, we consider three different scenarios. In the first scenario, conventional cellular communication takes place that does not involve D2D mode and clustering. The other two cases consider the proposed clustering algorithm, explained by the following text.

- Clustered D2D users with no interest factor

In this case, we assume that users do not share a common interest i.e., all of them are not interested in a single file (content). Users demand files of different sizes varying from 10 to 100 kB in a random manner. Though this scenario does not consider the social-factor, we still clustered them, as the literature suggests that even without the social-factor, clustering yields significant throughput gains [3,8,11,23]. The clustering criteria for these nodes are the same as mentioned in Equation (2) except that the interest-factor “*I*” is not considered. The weights selected are as follows: $w_{1}=\;0.4,\;w_{2}=\;0.6$. These were empirically selected to maximize throughput performance.

- Clustered D2D users with interest factor

The third scenario considers the social interest i.e., all the users in a given cluster are interested in a single file of size 100 kbits. This emulates social gatherings such as a concert or a stadium, where there is a large gathering, interested in a similar video/content. This scenario was implemented using the proposed algorithm. The value of the two weights remains the same as discussed in the previous scenario.

**Figure 8:**
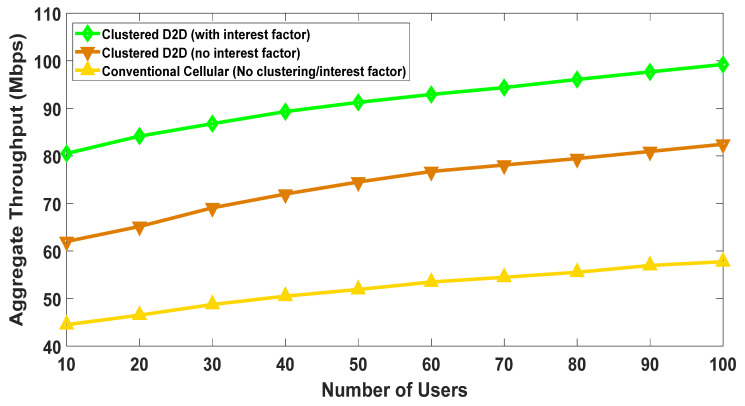
The impact of clustering and social-interest on throughput.

Figure 8 shows the result of aggregate throughput versus the number of users. It clearly shows the impact of social awareness as the aggregate throughput was maximum when it was considered. On the other hand, aggregate throughput was considerably low when social awareness was ignored. At the user density of one hundred, the difference between the two curves was approximately 19%. The throughput for a conventional cellular network with no clustering remained considerably low compared to the other two scenarios. This result, therefore, shows that clustering does play a vital role in enhancing the system’s performance. Furthermore, it can be seen that both social-interest and physical parameters (e.g., spatial distribution and channel gains) should be considered while modelling a system as it may bring significant benefits for the users as well as the whole network.

### 5.2. Benchmarking against Existing Algorithms

We selected five algorithms to benchmark against. Three of these are classical algorithms that are widely found in the literature, namely K-Medoids, Fuzzy C-Means (FCM), and Genetic Algorithm (GA) based clustering. These three algorithms have not been investigated and benchmarked within the context of D2D clustering and content-sharing applications, though an initial investigation was performed in our previous work [13]. The remaining two are the state-of-the-art and recently proposed algorithms. “Benchmarked I” has been proposed by Tulu.M.M et al. [69]. This algorithm applies the concept of entropy of betweenness centrality (EBC) to select CHs for content-sharing. The entropy of betweenness is based on the social relationship between the nodes and the shortest paths that exist between the nodes. “Benchmarked II” is proposed by Kazez C.A et al. [70]. This algorithm takes the neighbors and distance among the users as inputs for the selection of CHs.

#### 5.2.1. Throughput Comparison

**Figure 9:**
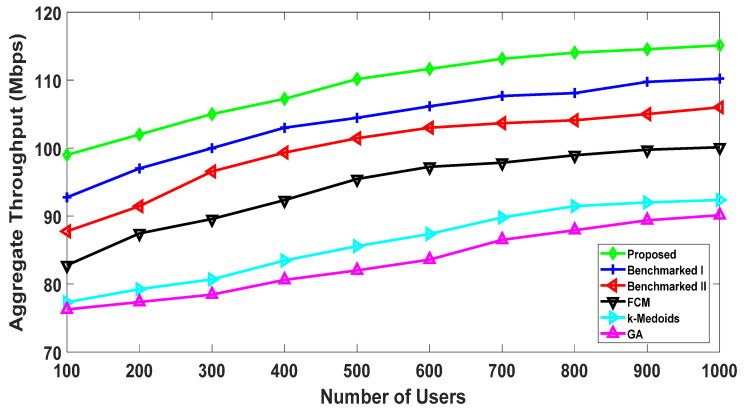
Aggregate throughput: comparison with the benchmarked ($w_{1}=\;0.4,\;w_{2}=\;0.6$).

The following result shows the comparison of the throughput performance. The proposed algorithm utilizes the social interest and physical parameters of the users to enhance the system’s performance. This was discussed in the previous result, and it is further elaborated in Figure 9, as it demonstrates that the proposed algorithm performs approximately 7% better than the next best algorithm (Benchmarked I) at one thousand nodes. Benchmarked algorithms I and II utilize the social interest, but they do not consider both distance and channel conditions among the users for clustering the users. Our result shows that consideration of both metrics does have a positive effect on the system’s throughput. This is because many users that are in proximity to each other may not have better channel conditions due to various factors (e.g., shadowing).

#### 5.2.2. Energy Consumption of Users

**Figure 10:**
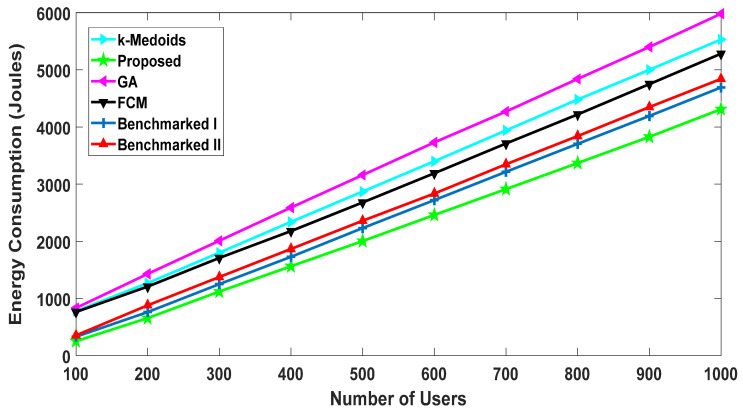
Energy consumption: comparison with the benchmarked ($w_{1}=\;0.4,\;w_{2}=\;0.6$).

The result shown in Figure 10 represents the energy consumption of the nodes in Joules with a varying number of users. It is evident from Figure 9 that we achieved better throughput as compared to the rest of the algorithms. If the file size of 100 kbits is constant, then the energy consumptions will be significantly dependent on the transfer rate. Consequently, the proposed algorithm performed the best (demonstrated by least energy consumptions) at different user densities as compared to the other algorithms. At one thousand nodes, the proposed algorithm approximately consumed 6% less energy as compared to the second-best algorithm.

**Figure 11:**
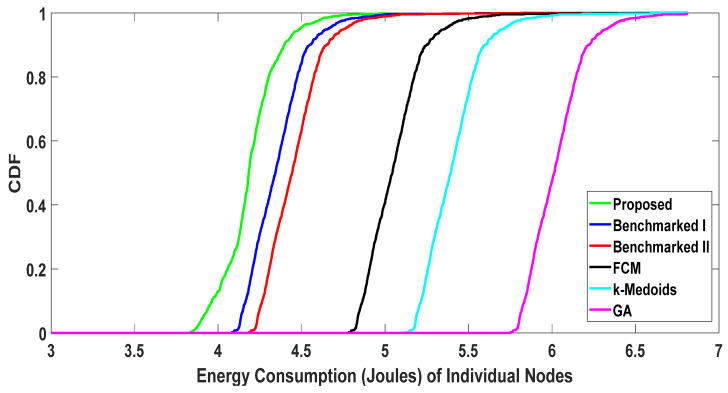
Cumulative Distribution Function (CDF) of energy consumption ($w_{1}=\;0.4,\;w_{2}=\;0.6$).

The energy consumption of the proposed algorithm is further elaborated in Figure 11. The Cumulative Distribution Function (CDF) of the energy consumption is presented for the proposed algorithm and the benchmarked clustering algorithms at a user density of one thousand. We can observe that even at the node level, the proposed algorithm outperformed the benchmarked algorithms in all quartiles with regard to energy consumption. Therefore, the overall lower energy consumption was not achieved by favoring a few nodes to a large extent while disregarding the others.

#### 5.2.3. Area Spectral Efficiency

**Figure 12:**
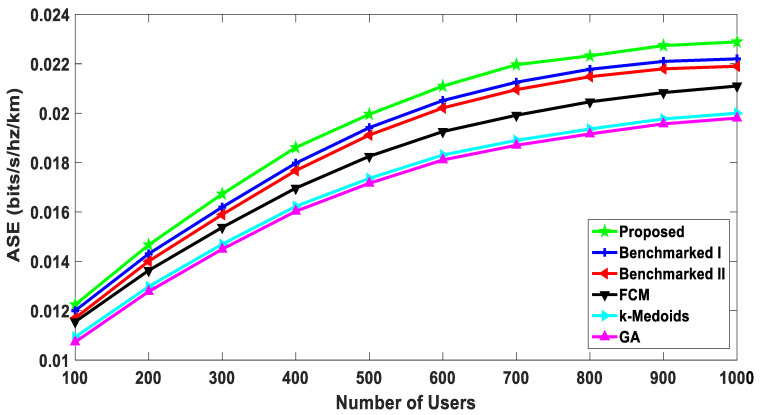
ASE: comparison with the benchmarked ($w_{1}=\;0.4,\;w_{2}=\;0.6$).

Area Spectral Efficiency represents the sum of average achievable rates per unit bandwidth per unit area [71]. To the best of the authors’ knowledge, ASE has not been evaluated for all the five benchmarked algorithms. It can be observed in Figure 12 that the ASE of the proposed algorithm was better than all the benchmarked algorithms. ASE depends significantly on average rates of the users if the area and per unit bandwidth remain constant. Therefore, the proposed algorithm has higher ASE. Furthermore, the performance was better than all the other benchmarked schemes. It is also encouraging to observe that the performance improved for the proposed algorithm as the user density increased. This shows the scalability of the proposed algorithm. The proposed algorithm showed approximately 3% improvement in ASE at the node density of one thousand, as compared to the benchmarked scheme I that showed the second-best performance. The classical algorithms for generic clustering are not purpose-built for a D2D scenario and are far inferior to the proposed, “Benchmarked I” and “Benchmarked II” algorithms.

### 5.3. The Optimal Number of Clusters

We investigated the effect of the number of clusters on the energy consumption and ASE. It has not been reported in the literature considering D2D Content-Centric Networks. The selection of the number of clusters significantly affects the clustering performance. A trade-off always exists when it comes to selecting the number of clusters. Increasing the number of clusters up to a certain extent will bring benefits but at the expense of increased signaling and complexity. The clustering metrics that we selected for the proposed algorithm can vary significantly; thus, it is not easy to predetermine the cluster size. Therefore, the size of the cluster is variable.

However, there should be a criterion that can help determine the number of clusters that can be formed based on a given scenario such as user distribution, values of the clustering metrics, etc. In this study, the Calinski–Harabasz (Cal–Har) criterion [66] was selected. It is also termed as the variance ratio criterion. Mathematically, it can be defined as:

(12) $$Cluster\;Size_{\left(Cal-Har\right)}=\;\frac{V_{B}}{V_{W}}*\frac{\left(N-C\right)}{\left(C-1\right)}$$

In Equation (12), between-cluster and within-cluster variance are represented by $V_{B}$ and $V_{W,}$ respectively. The total number of users is denoted by N, whereas C is the number of clusters against which this criterion will be judged. Clustering metrics determine the variance between and within clusters. To find the optimal solution, Equation (12) needs to be maximized with respect to the number of clusters. As the ratio of the variances given in Equation (12) increases, user segregation becomes more precise which leads to the optimal number of clusters.

**Figure 13:**
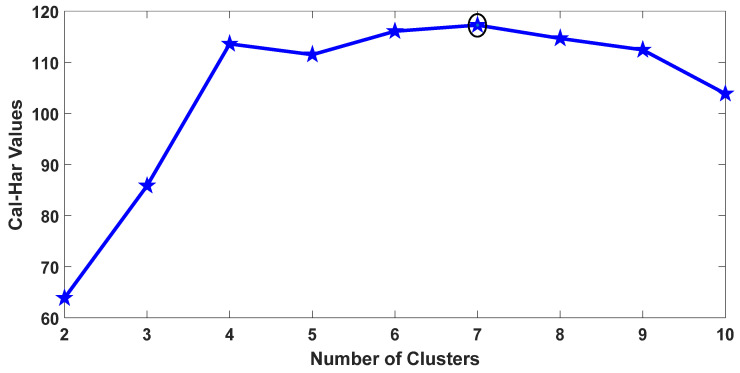
Optimal number of clusters: Cal–Har criterion.

**Figure 14:**
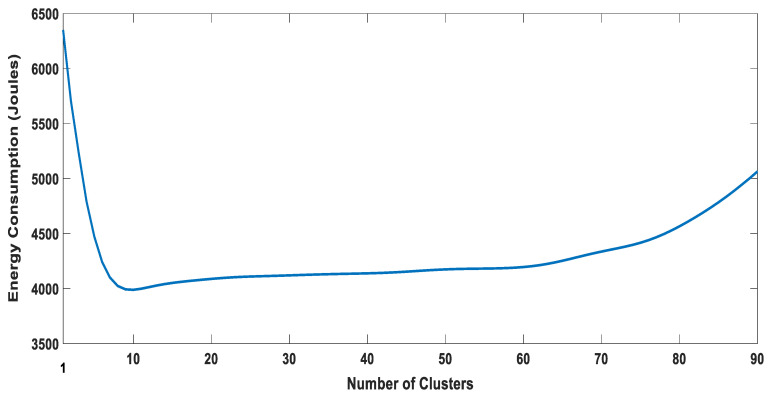
Energy consumption and the number of clusters: a comparison.

**Figure 15:**
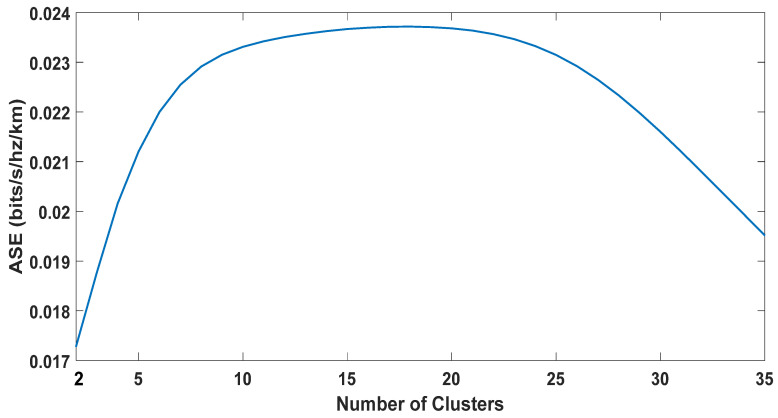
ASE and the number of clusters: a comparison.

For a user density of one thousand, the Cal–Har criterion result is depicted in Figure 13. The criterion value was highest when the number of clusters was seven. Therefore, for the given user distribution and node density, the optimal number of clusters should be seven. We then investigated whether this is the optimal choice when considering energy consumption, and ASE. Results for both parameters for a various number of clusters are presented in Figure 14 and Figure 15, respectively. A trade-off can be seen for the choice of the number of clusters. As shown in Figure 14, the result for energy consumption aligns with the Calinski–Harabasz criterion, as the lowest energy consumption occurred when the number of clusters was seven to ten. On the other hand, the optimal number of clusters for ASE appears to lie between 10 and 15.

It is not straight forward to suggest a certain number of clusters based on the above-mentioned results. It is important to consider a few additional factors that might influence the selection. The signaling overhead and complexity of cluster maintenance increase with the increase in the number of clusters. Moreover, we can observe from the results presented in Figure 14 and Figure 15 that ASE did not differ significantly when forming seven to ten clusters as opposed to forming 10–15. Therefore, seven is likely to be a better choice, since it represents the lowest energy consumption while sacrificing minimal ASE gain for lower signaling overhead and complexity.

Various external factors influence the choice of the number of clusters as well. As discussed earlier, our study considers forming clusters only for those users that are interested in content sharing. Therefore, in some situations, only a few users might be interested in content-sharing, and hence, making a certain number of clusters is not necessary. On the other hand, a scenario can build-up where a large group of users is interested in content-sharing, but even in this case, the physical location of the users might influence the choice of the number of clusters. A large number of closely packed users at a concert or a sports event only needs a few clusters. As opposed to this scenario, users sharing a common interest might be dispersed in a geographical area, requiring a higher number of clusters. More clusters can also be formed in a scenario where users might be in close vicinity, but they have different content of interest. In that case, users sharing the same interest are expected to be in one cluster, whereas the rest of the users should form a separate cluster. Moreover, some users may be better served by the BS, and it should not be mandatory for all the users to be considered for the clustering.

### 5.4. Throughput Fairness

The benchmarked algorithms considered in this study do not consider throughput fairness as a potential performance parameter. The literature suggests that fairness is crucial for evaluating a cellular network [72,73]. Therefore, we extended the proposed algorithm to suit the throughput fairness as well. To target the fairness, we introduced another clustering metric—the variance among the channel conditions of the users.

Considering only absolute channel conditions might disregard many users having not so good channel conditions. Therefore, in that case, there would be a significant difference in the throughputs of the individual users. Owing to this reason, we introduced variance of channel conditions, and our assumption is validated by the results demonstrating throughput fairness, shown later in this section.

Moreover, since we are considering a multicasting scenario, we are looking for approximately similar channel conditions with each node. The reason being, in a multicasting scenario, if each device receives the transmission at significantly different rates, then the complexity of the system would increase and might become infeasible [74].

Let us take an example of a scenario where a video stream needs to be broadcasted to a group of users. Assume that there are a few users with higher rates as compared to the others. Since we are considering broadcasting, the maximum achievable rates are determined by the worst physical link in the group. Therefore, users having higher rates will face long delays waiting for the other users to catch up. This reason makes it even more significant to have variance in channel conditions as an important factor in addition to just the absolute channel conditions. Though, owing to the degradation in the throughput performance, variance in channel conditions cannot be selected as the sole criterion. Therefore, the modified clustering algorithm considers all three different metrics which are attached to their respective weights.

The introduction of variance among channel conditions modifies the Equation (2), to the following;

(13) $$W_{T}=I.\left[w_{1}*D_{\left(a,b\right)}+\;w_{2}*\left(\frac{1\;}{h_{ab\;}}\right)+w_{3}*var\left(h\right)\right]$$

$w_{3}$ represents the weights given to variance of the channel gains. The term “Var(h)” represents the variance among the channel conditions of the users. The weights represented in Equation (13) are such that $\sum_{f=1}^{3}w_{f}=1$. The node with the minimum $W_{T}$ is chosen as the CH.

Jain’s fairness model [75] was used to evaluate the fairness performance of the proposed algorithm. The Jain’s fairness index denoted by J(x) is represented by Equation (14).

(14) $$J\left(x\right)=\;\frac{{\left(\sum_{i=1}^{M}x_{i}\right)}^{2}}{M\sum_{i=1}^{M}x_{i}{}^{2}}$$

$x_{i}$ represents the throughput of the ith user, given that there are total M users.

**Figure 16:**
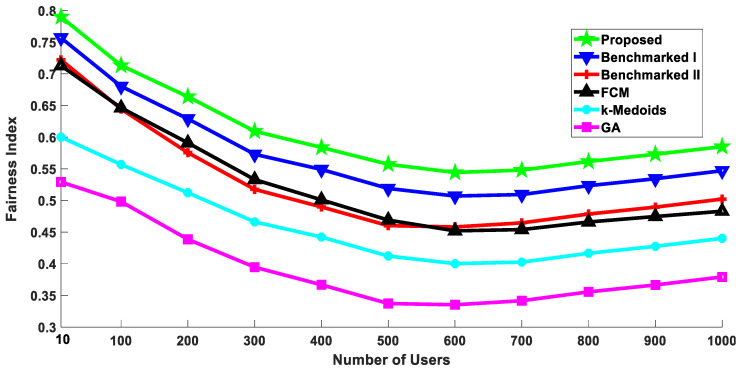
Jain’s Fairness Index: Comparison with the benchmarked ($w_{1}=w_{2}=0.1,\;w_{3}=0.8$).

We simulated the proposed algorithm with the weights as follows: $w_{1}=w_{2}=0.1,\;w_{3}=0.8$. The weights selected for this result were empirically adjusted to enhance fairness. So, the maximum weight is given to the variance of channels. Though the selected weights did not yield the best energy consumption and ASE, it did outperform all the benchmarked algorithms when it comes to fairness. This is depicted in Figure 16. At the user density of one thousand, the proposed algorithm performed approximately 7% better than the benchmarked scheme I, which performed the best among the existing algorithms. This result demonstrates the flexibility of the proposed algorithm. By simply adjusting the weights, it is possible to achieve better fairness. It should be noted that the works reporting the benchmarked algorithms I and II do not discuss fairness. These algorithms also do not have any parameter to perform this trade-off.

### 5.5. The Trade-Off among Different Performance Parameters

**Figure 17:**
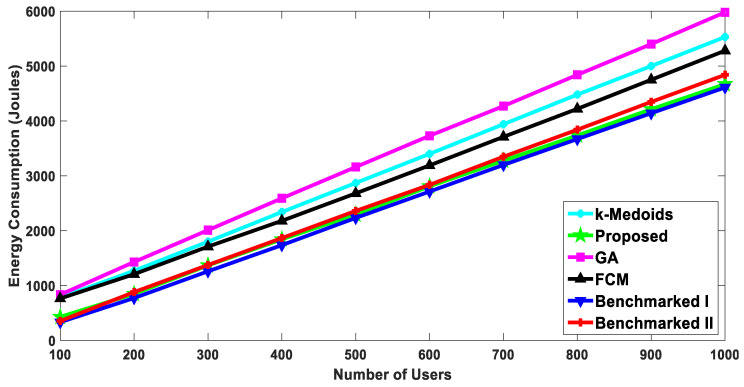
Energy consumption: comparison with the benchmarked schemes ($w_{1}=w_{2}=0.1,\;w_{3}=\;0.8$).

**Figure 18:**
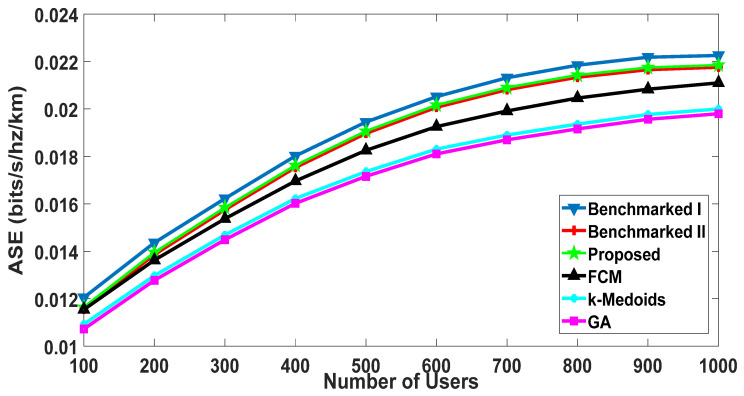
ASE: comparison with the benchmarked schemes ($w_{1}\;=\;w_{2}=\;0.1,\;w_{3}\;=\;0.8$).

The energy consumption and ASE for the weights $w_{1}=w_{2}=0.1\;\mathrm{and}\;w_{3}=0.8$ are presented in Figure 17 and Figure 18, respectively. It can be observed that the cost of improved fairness is a slight degradation in performance with regard to energy consumption and ASE. However, the performance of the proposed algorithm is satisfactory in the sense that it is better than the other four benchmarked algorithms and there is only a very small performance gap with the best scheme. The proposed algorithm is able to trade-off energy consumption and ASE with fairness, which is not possible in any of the benchmarked algorithms. Our algorithm provides this flexibility by adjusting the weights to enhance the desired performance parameter accordingly. It can achieve the best performance for a given parameter while providing a satisfactory performance with respect to the rest.

To the best of the author’s knowledge, the algorithms considered for benchmarking in this study have not been investigated for all the three performance parameters i.e., energy consumption, ASE, and fairness.

## 6. Conclusions

This paper presented a content-sharing framework for D2D communication in a multicasting scenario. We utilize Content-Centric Networking and Network Virtualization to propose a distributive architecture. This study showed the significance of spatial distribution and social-ties on the throughput’s performance and established that both are vital for enhancing the performance of the Content-Centric Network.

A novel weighted clustering algorithm was incorporated in the proposed architecture. It is evident from the results that clustering enhanced the system’s performance. The performance of the proposed algorithm was thoroughly investigated against different popular clustering algorithms. The proposed algorithm shows a 6% improvement in energy consumption while achieving 3% better ASE as compared to the best-benchmarked algorithm. The effect of the number of clusters on the energy consumption of users and ASE was also investigated. A trade-off exists between the two metrics in the selection of the number of clusters. The optimal energy consumption was achieved at a smaller number of clusters as compared to ASE. It is suggested that signaling overhead required to set up more clusters should be considered while selecting the number of clusters. Hence, the formation of a smaller number of clusters showing optimal energy consumptions at the cost of marginal degradation in ASE is acceptable. A slight modification in the algorithm and weight adjustment improved throughput fairness up to 7%. On the contrary, all the benchmarked algorithms do not consider fairness.

For future research, it would be important to apply machine learning algorithms to optimally select the weights according to the system’s requirements. The authors also intend to investigate the performance of the proposed scheme for various multimedia applications. Additionally, the comparative study of the signaling overhead required for the proposed algorithm needs to be explored as well. Moreover, while this study provides an approach to finding the number of clusters, one of the future directions could be a comprehensive study of optimal selection of the number of clusters.

## Figures and Tables

**Table 1:** Summary of related research articles.

Research	Year	Distributed Architecture	Performance Parameters			
			Throughput	Energy Consumption	ASE (Area Spectral Efficiency)	Fairness
Asadi et al. [34]	2016	×	✓	✓	×	✓
Zhang et al. [35]	2017	×	×	✓	×	×
Yang et al. [12]	2018	×	×	✓	×	×
Huang et al. [36]	2018	×	✓	×	×	×
Rahman et al. [37]	2018	×	×	✓	×	×
Pizzi et al. [38]	2019	×	✓	×	×	×
Aslam et al. [13]	2019	×	✓	✓	✓	×
Shi et al. [39]	2019	×	×	✓	×	×
Wu et al. [40]	2019	×	✓	×	×	×
Wang et al. [41]	2019	×	×	×	✓	×
Zhou et al. [42]	2020	×	×	✓	✓	×
Our Proposal	2020	✓	✓	✓	✓	✓

**Table 2:** List of Symbols.

Symbol	Representation
N	Set comprises of all the users
k	Index of cluster member
CH	Cluster Head
$R_{CH_{j}}$	Achievable Rate of $CH_{j}$ when receiving the contents from the Base Station (BS)
$SNR_{CH_{j}}$	Signal-to-Noise Ratio of a $CH_{j}$
$N_{o}$	Noise Spectral Density
B	Bandwidth of the Transmission Channel
$h_{BS,CH_{j}}$	Channel Gain between the BS and the $CH_{j}$
$P_{B}$	Transmit Power of the BS
$R_{m_{k}}$	Achievable Rate of cluster member $m_{k}$
$h_{m_{k},CH_{j}}$	Channel Gain between the cluster member $m_{k}$ and $CH_{j}$
$P_{CH_{j}}$	Transmit Power of the $CH_{j}$
$F_{S}$	File Size (size of the demanded content)
$P_{chrx}$	Power consumed by the CH to receive the contents from BS
$P_{mrx}$	Power consumed by the cluster member to receive the content from cluster head (CH)

**Table 3:** Simulation parameters.

Parameters	Value
Simulation Platform	MATLAB
Channel Model	Rayleigh Distributed
User Placement	Uniformly Distributed
Node Density	100 to 1000
Cluster Size	Variable
Number of Clusters	Variable
Transmit Power of CH	1.425 Joules/s
Power required to receive data from BS	1.8 Joules/s
Power required to receive data from CH	0.925 Joules/s
Content Considered	A file of size 100 kBits
Classical benchmarked Schemes	K-Medoids (KM), Genetic Algorithm (GA) and Fuzzy C-Means (FCM)
State-of-the-art benchmarked Schemes	Proposed in [69]. (referred in this document as benchmarked I) Proposed in [70]. (referred in this document as benchmarked II)
Number of Simulation Runs	10,000
